# Isolation, structures, bioactivities, application and future prospective for polysaccharides from *Tremella aurantialba*: A review

**DOI:** 10.3389/fimmu.2022.1091210

**Published:** 2022-12-08

**Authors:** Yonghuan Yan, Mengtian Wang, Ning Chen, Xu Wang, Chenghao Fu, Yuemin Li, Xiaoruo Gan, Pin Lv, Yan Zhang

**Affiliations:** ^1^ School of Forensic Medicine, Hebei Key Laboratory of Forensic Medicine, Hebei Medical University, Shijiazhuang, China; ^2^ Hebei Food Inspection and Research Institute, Hebei Food Safety Key Laboratory, Key Laboratory of Special Food Supervision Technology for State Market Regulation, Hebei Engineering Research Center for Special Food Safety and Health, Shijiazhuang, Hebei, China; ^3^ Department of Cell Biology, Cardiovascular Medical Science Center, Key Laboratory of Neural and Vascular Biology of Ministry of Education, Hebei Medical University, Shijiazhuang, China

**Keywords:** *tremella aurantialba*, polysaccharides, isolation, structures, bioactivities, application

## Abstract

Since ancient times, *Tremella aurantialba* has been proposed to have medicinal and food benefits. Modern phytochemistry and pharmacological studies have demonstrated that polysaccharides, the main components from *T. aurantialba* appear to be an all-round talent resisting a variety of chronic inflammatory diseases and protecting against different types of tumors, diabetes and cardiovascular diseases. These health and pharmacological benefits have gained much attention from scholars around the world. Further, more and more methods for polysaccharides extraction, purification, structure identification have been proposed. Significantly, the bioactivity of fungus polysaccharides is affected by many factors such as extraction and purification conditions and chemical structure. This paper provides an overview of recent advances in the isolation, structural features and biological effects of polysaccharides derived from *T. aurantialba*, covers recent advances in the field and outlines future research and applications of these polysaccharides.

## 1 Introduction

**Figure 1:**
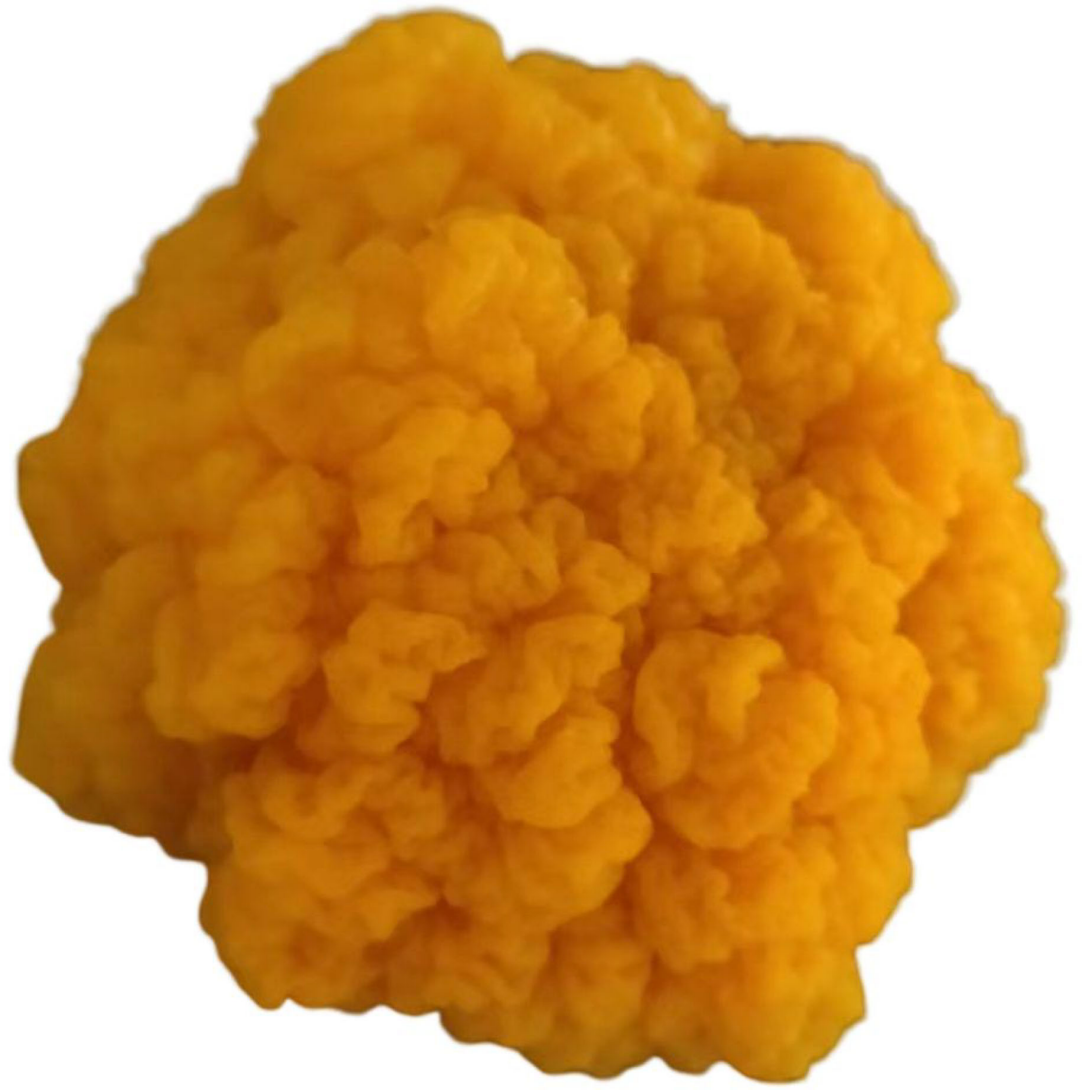
The fruiting bodies of *T. aurantialba*.

*Tremella aurantialba*, belonging to the genus *Naematelia Fr*, is a well-known medicinal and edible fungus widely distributed in Asia, Europe, North America and Oceania (1–3). It is now found all over the world due to artificial cultivation. The color is golden yellow, the taste is delicate and sticky, and the nutritional value are better than those of *Tremella Fuciformis* and *auricularia auricula* (4). Relevant pictures of *T. aurantialba* are shown in **Figure 1**. Wild *T. aurantialba* have been consumed as food and medicine in countries such as Asia and Europe for thousands of years (2, 5). It was once considered to have the functions of “benefiting the mind and body”, “turning the weak into the strong” and “prolonging life” (6). Besides, the Chinese medicine book “Compendium of Materia Medica” records that *T. aurantialba* can be used to treat a variety of diseases, especially moistening the lung, relieving cough, protecting the liver and tonifying the kidney. Xizang Common Chinese Herbal Medicine also records the effects of *T. aurantialba* on asthenia tuberculosis cough, hemoptysis, tuberculosis, asthma, hypertension and chronic bronchitis in the elderly (7).

In recent decades, the fungus polysaccharides as ideal natural resources for supplements and pharmaceuticals have attracted a lot of attention due to their abundant bioactivities (8–12). Modern phytochemistry and pharmacological studies have been proved that polysaccharides are major bioactive constituents of *T. aurantialba*, accounting for 37.8%-40.55% (13). Presently, crude polysaccharides or their purified fractions from *T. aurantialba* are attracting growing attention due to their remarkable immune-stimulating activity (4, 14) and the ability to resist various chronic inflammatory diseases (15–18). However, there are few published reviews concerning extraction, purification, structure and biological activities of polysaccharides from *T. aurantialba*. Based on current available studies, it is speculated that *T. aurantialba* may be used as more promising functional food supplements or therapeutic agents than some other fungi such as *Auricularia auricula* (4, 19). Therefore, this paper aims to systematically summarize and update some of the latest research achievements of *T. aurantialba* polysaccharides, including extraction, purification, structural characterization, biological activity and the relationship between its structure and activity, so as to provide detailed reference for the future utilization of *T. aurantialba* polysaccharides.

## 2 Procedure of extraction and purification

Polysaccharides are the structural components of the fungus cell wall. Therefore, it is critical to select an efficient extraction method that can destroy the cell wall to release fungus polysaccharides (20). At present, the methods used for extraction of *T. aurantialba* polysaccharides include hot water extraction, alkali extraction, enzyme-assisted extraction and ultrasonic-assisted extraction.

### 2.1 Extraction

#### 2.1.1 Hot water extraction

Hot water extraction is the most classical and convenient method in the laboratory and industrial extraction of fungus polysaccharides (2, 18, 21). It is often used in combination with other methods such as hydrolysis (alkali (22–25) and enzyme (26–30)), chemical derivatization (31–36), or physical treatment (37–41) to obtain polysaccharides with specific structural features and high bioactivity. For example, for some acidic or high molecular weight polysaccharides, especially those containing uronic acids, their solubility in dilute alkali solution is generally greater than that in hot water, so alkali extraction of such polysaccharides is common. Dong compared the effects of water extraction and alkaline extraction on the polysaccharides from *T. aurantialba* mycelium, and the results showed that when the latter method was used, the extracted mycelium polysaccharides content increased by 9.4% (42). Liu and Yu compared the polysaccharides yield obtained by hot water extraction and alkali extraction, and found that the polysaccharides yield of alkali extraction reached 14.9% at 4 h after treatment, and then showed a downward trend. The yield of hot water extraction was 10.3% at 4 h, and the yield reached the maximum at 10 h, which was 15.5%. The results showed that under suitable conditions, alkaline extraction could increase the yield of polysaccharides, but might also cause polysaccharides degradation (43).

#### 2.1.2 Enzymatic extraction

Enzymatic extraction is also commonly used to increase the yield of polysaccharides. Wang compared the effects of water extraction, enzymatic extraction, microwave extraction and ultrasonic assisted extraction on polysaccharides yield of *T. aurantialba* fruiting bodies. The results showed that under the optimal extraction conditions, enzymatic extraction obtained the highest polysaccharides yield, up to 25.96%, with the highest antioxidant activity (44).

#### 2.1.3 Ultrasonic assisted extraction

Ultrasonic assisted extraction method has gradually become a popular method for the extraction of various fungus polysaccharides in recent years. Ultrasound can destroy the structure of fungus cell walls and make polysaccharides flow out of cells faster, thereby effectively shortening the extraction time of polysaccharides and improving the yield of polysaccharides. Huang et al. compared the effects of water extraction and ultrasonic assisted extraction on the yield of polysaccharides from *T. aurantialba* mycelium, and found that compared with water extraction, ultrasonic assisted extraction had a higher polysaccharides yield (45). However, when the ultrasonic power exceeded 600 W, the polysaccharides yield would decrease, which might be related to the ultrasonic-induced polysaccharides degradation.

#### 2.1.4 Others

Several new polysaccharides extraction methods, including microwave extraction (41, 46–49), pressurized liquid extraction (49–52), ultrasound-enhanced subcritical water extraction (53–56), continuous fractionation by supercritical fluid extraction (57) and induced or pulsed electric fields extraction (58, 59) have been proposed for efficient extraction of polysaccharides. These deserve further attention in the extraction process of *T. aurantialba* polysaccharides.

In addition, extraction conditions, including extraction solvent, the ratio of raw material to solvent, time, temperature, etc., all have a great influence on the yield of polysaccharides. In this regard, Huang used a single factor test to improve the extraction rate of *T. aurantialba* polysaccharides. The optimized conditions were as follows: the ratio of water to mycelium powder was 120: 1 (V/W), 100 °C, ultrasonic power 600 W, extraction for 20 min and extraction twice. Under these conditions, the maximum yield of *T. aurantialba* polysaccharides was 11.16% (45). Jiang et al. adopted the Box-Benhnken method to investigate the ultrasonic-assisted extraction parameters of *T. aurantialba* polysaccharides. It was concluded that under the best conditions of ultrasonic power of 518 W, ultrasonic time of 16 min and ultrasonic temperature of 50 °C, the extraction amount of *T. aurantialba* polysaccharides could reach 2.85 g/L (60).

### 2.2 Purification

The primary extracted crude polysaccharides usually contain many impurities. In order to fully explore the pharmacological activities of *T. aurantialba* polysaccharides, the crude polysaccharides extracted initially are usually further collected and purified to obtain pure polysaccharides. At present, the purification methods for *T. aurantialba* polysaccharides include ethanol fractional precipitation, fiber membrane filtration, gel chromatography, anion exchange column chromatography and gel permeation chromatography. Among them, hydro-alcohol precipitation method is a routine method for isolating and purifying fungus polysaccharides. Besides, some studies have combined the various methods mentioned above to prepare pure polysaccharides from *T. aurantialba* in recent years. For example, Du et al. used ultrafiltration, anion exchange column chromatography and gel permeation column chromatography to separate and purify the crude polysaccharides from *T. aurantialba* and finally obtained two acidic heteropolysaccharides, TAPA1 and TAPB1 (2, 61). Deng et al. used microfiltration and gel permeation column chromatography to separate and purify polysaccharides from *T. aurantialba* fermentation broth (62). The table below gives a summary of the methods for extracting and purifying polysaccharides from *T. aurantialba* (**Table 1**). It is worth noting that *T. aurantialba* also contains high levels of proteins, pigments and other molecules, which may affect the extraction and activity of polysaccharides. Thus, some organic solvents such as petroleum ether (69) and ethanol at different concentrations (2, 61, 64) are commonly used to remove interfering components such as pigment, fat, monosaccharide and disaccharide before extraction. Additionally, savage reagent is often used to remove proteins from extracts prior to characterization to obtain crude polysaccharides (70). Unfortunately, this method is inefficient, complex, and time-consuming, and the use of organic solvents (n-butanol and chloroform) also adversely affects the purity of the synthetic products and the environment (71). Therefore, proteolytic enzymes have been proposed to remove protein components during polysaccharides purification (72).

**Table 1:** **A** summary of the methods of extraction and purification, conditions and total yield of polysaccharides from *T. aurantialba*.

Sources	Extraction methods	Processing conditions			Enzymatic conditions					Total yield (%)	Isolation and purification method	Ref.
		Time	Temperature/Power	Solid-liquid ratio (mL/g)	Enzyme	Enzyme dosage	Temperature	Time	pH			
Fruiting bodies	HWE	2 h×3	100 °C	10: 1	–	–	–	–	–	–	Ultrafiltration, anion-exchange chromatography, gel-permeation chromatography	(61)
Fruiting bodies	HWE	2 h×3	100 °C	10: 1	–	–	–	–	–	–	Ultrafiltration, anion-exchange chromatography, gel-permeation chromatography	(2)
Fruiting bodies	HWE	3 h×3	100 °C	10: 1	–	–	–	–	–	3.84	Ultrafiltration, anion-exchange chromatography, gel chromatography	(63)
Fruiting bodies	HWE	3 h×3	95 °C	40: 1	–	–	–	–	–	50.6	Anion-exchange chromatography	(4)
Fruiting bodies	HWE	10 h	100 °C	–	–	–	–	–	–	8	Gel-permeation chromatography	(64)
Fruiting bodies	HWE	3 h	60 °C	41: 1	–	–	–	–	–	7.9	–	(65)
Fruiting bodies	EAE	3 h	65 °C	25: 1	Cellulase, pectinase, papain 1: 1: 1	6 g/L	45 °C	3 h	–	11.59 ± 0.10	–	(66)
Fruiting bodies	EAE	52 min	52 °C	347: 1	Pectinase, cellulase 6: 4	20.50 g/L	52 °C	52 min	–	12.69 ± 0.52	–	(67)
Fruiting bodies	EAE	1 h	80 °C	80: 1	Cellulase, papain, pectinase 4: 4: 3	6.7% (w/w)	45 °C	45 min	–	28.16	Stepwise alcohol precipitation	(44)
		1 h	80 °C	80: 1	Cellulase	6.7% (w/w)	60 °C	60 min	3.51	25.96	Stepwise alcohol precipitation
Fruiting bodies	HWE	3 h	65 °C	25: 1	–	–	–	–	–	8.02	–	(45)
Fruiting bodies	EAE	4 h×3	100 °C	15: 1	Cellulase, pectinase, papain	6% (w/w)	55 °C	45 min	4.5	–	–	(1)
Mycelium	HWE	4 h×3	100 °C	10: 1	–	–	–	–	–	–	Stepwise alcohol precipitation	(21)
Mycelium	ABE	4-6 h	25 °C	–	–	–	–	–	–	–	Gel-permeation chromatography	(14)
Mycelium	ABE	4 h	40 °C	25: 1	–	–	–	–	–	–	–	(42)
	HWE	3-5 h×4	70-80 °C	5: 1	–	–	–	–	–	–	–
Mycelium	UAE	20 min×2	600 W	120: 1	–	–	–	–	–	11.16	–	(45)
	HWE	2 h×2	100 °C	160: 1	–	–	–	–	–	7.27	–
Mycelium	HWE	6 h×4	80 °C	10: 1	–	–	–	–	–	–	–	(18)
Fermentation solution	ABE	4 h	40 °C	–	–	–	–	–	–	–	Gel chromatography	(62)
Fermentation solution	HWE	30 min×3	100 °C	–	–	–	–	–	–	14.3	–	(68)
Fermentation solution	UAE	16 min	518 W 、50 °C	–	–	–	–	–	–	–	–	(60)
Fermentation solution	HWE	10 h	98-100 °C	20: 1	–	–	–	–	–	15.5	–	(43)
	ABE	4 h	90 °C	20: 1	–	–	–	–	10	14.9	–

HWE, hot-water extraction; UAE, ultrasound-assisted extraction; EAE, enzyme-assisted extraction; ABE, alkali-base extraction.

## 3 Physiochemical and structural features

A large number of studies have shown that the biological activities of fungus polysaccharides depend on their complex physiochemical properties. The molecular weight, chain length, type and number of functional groups, type of glycosidic bond and triple helix structure of polysaccharides all have significant effects on their biological activities. Therefore, structural identification of polysaccharides and subsequent structure-activity relationship analysis are very important, but also a challenge. To date, the identification of *T. aurantialba* polysaccharides has focused on their physicochemical properties (purity, molecular mass and distribution) and structural characterization (composition of monosaccharides and ratios, characteristics of glycosidic bonds). Herein, the reported *T. aurantialba* polysaccharides in the past few years are listed and integrated information about their source, molecular weight, monosaccharide composition and structures are comprehensively introduced in **Table 2**. Many technologies including infrared (IR) spectroscopy, ultraviolet (UV)-visible spectroscopy, high-performance anion-exchange pulsed amperometric detection chromatography (HPAEC-PAD), gas chromatography-mass spectrometry (GC-MS), nuclear magnetic resonance (NMR) spectroscopy, high-performance liquid chromatography (HPLC), gas chromatography (GC), etc. have been used for determining the primary structures of *T. aurantialba* polysaccharides and scanning electron microscopy have been used to analyze the advanced structure of *T. aurantialba* polysaccharides. It is worth noting that gel permeation chromatography technology (GPC) is widely applied to evaluate the molecular weight of *T. aurantialba* polysaccharides and GC, PMP pre-column derivatization HPLC or high-performance anion-exchange pulsed amperometric detection chromatography is used for the monosaccharide composition analysis. Since polysaccharides generally do not have UV or fluorescent chromophore groups, derivatization is necessary for structural identification of *T. aurantialba* polysaccharides by GC or HPLC. The complex structure and derivatization of polysaccharides may lead to the loss of bonds or the emergence of isomers. In the future, it is still necessary to continuously improve the structure identification technology of polysaccharides. Furthermore, although many polysaccharides with different structural characteristics have been obtained from *T. aurantialba*, only a small amount of structural information has been published. Further research should be carried out in combination of advanced techniques to clarify the primary structure of *T. aurantialba* polysaccharides.

**Table 2:** **A** summary of the structure of polysaccharides obtained from *T. aurantialba*.

Sources	Carbohydrate content	Name	Characterization	Molecular weight (kDa)	Monosaccharide analysis	Structural characterization	Analytical techniques		Bioactivities	Ref.
Fermentation solution	–	–	–	–	Gal, Rha, Xyl, Ara, Glc, Fuc	(1→3), (1→4) and (1→6)-glycosidic bond, α, β-glycosidic bond	GFC, TLC, IR		–	(62)
Fermentation solution	–	LQP	–	14	Glc: Gal: Man: Xyl: Ara = 42.37: 39.47: 14.05: 3.74: 0.37	β-glycosidic bond	UV, GC-MS, GPC, FT-IR		Antioxidant, hypoglycemic	(15)
Fermentation solution	–	FTAP	Neutral polysaccharides	–	–	–	GFC		Hypoglycemic	(43)
Fermentation solution	–	TBE	–	–	–	–	TLC		Antioxidant, hypolipidemic	(21)
Fermentation solution	–	TAPS-F	–	2924.6	Man-N: Man: GlcA: Glc: Xyl = 3.3: 52.0: 4.1: 2.4: 1.8: 36.4	β-glycosidic bond	HPLC, UV, GPC, FT-IR		–	(1)
Fruiting bodies	–	TAPS-E	–	1130.4	Man-N: Man: GlcA: Glc: Xyl = 8.7: 47.4: 2.0: 3.4: 1.0: 37.5		HPLC, UV, GPC, FT-IR		–	
Fruiting bodies	–	TAP	Acidic heteropolysaccharide	1500	Man: Xyl: GlcA: Glc = 4: 2: 1: -0.3	(1→3) linked α-D-Manp backbone, C-2 linked (1→3)-β-D-Xylp and β-D-glucopyranosyl-uronic acid, C-4-terminal α-D-Manp	IR, PPC, GC, GFC		Hypoglycemic	(73)
Fruiting bodies	98.7%	TAPA1	Acidic heteropolysaccharide	1350	D-Man: D-Xyl: D-GlcA = 5: 4: 1	α-(1→3) linked Manp backbone, C-4-Xylp, C-2-side chains composed of either Xyl, Man, and GlcA or of Xyl and Man	GC-MS, FT-IR, GPC, HPAEC-PAD, NMR (^1^ H, ^13^ C), COSY, TOCSY, NOESY, HMQC, HMBC		Immunostimulatory	(61)
Fruiting bodies	97.6%	TAPB1	Acidic heteropolysaccharide	760	D-Man: D-Xyl: D-GlcA = 3.1: 2.9: 1.2	α-(1→3) linked Manp backbone, O-4-side chain composed of two Xyl, O-2-side chain comprising Xyl and GlcA	GC-MS, FT-IR, GPC, HPAEC-PAD, NMR (^1^ H, ^13^ C), COSY, TOCSY, NOESY, HMQC, HMBC		Antioxidant	(2)
Fruiting bodies	61.76 ± 1.7%	TAP-3	Acidic heteropolysaccharide	624	D-Man: D-Xyl: D-GlcA = 3: 1: 1	(1→3) and (1→2)-α-Manp backbone, C-2 -β-Xylp, β-GlcpA, C-6-acetyl groups	HPGPC, HPLC, FT-IR, GC-MS, NMR (^1^ H, ^13^ C), HSQC, ROESY, HMBC, COSY, TOCSY, SEM		Immunomodulatory	(4)
Fruiting bodies	–	TAP	Acidic heteropolysaccharide	–	D-Glc: D-Xyl: D-Man: D-GlcA = 22.50: 24.47: 46.29: 6.64	β-pyranose	HPAEC-PAD, FT-IR		Antioxidant	(44)
		TAP30			D-Glc: D-Xyl: D-Man: D-GlcA = 39.51: 10.95: 39.97: 11.28				
		TAP60			D-Gal: D-Glc: D-Xyl: D-Man: D-GlcA = 4.37: 34.86: 21.78: 56.04: 14.92				
		TAP80			D-Gal: D-Glc: D-Xyl: D-Man: D-GlcA = 6.92: 39.04: 25.45: 58.20: 10.87				
Fruiting bodies	–	JP-2	–	180	Man 、Glc 、Xyl 、Rha 、Gal	β- (1, 3) and β- (1, 4)-glycosidic bonds	UV, GFC, PPC, IR, ^13^ C NMR		–	(74)
Mycelium	–	MCP	–	43	D-Glc: D-Gal: D-Man: D- Rha: D−Ara: D−Xyl = 82.17: 9.89: 4.53: 1.31: 0.74: 1.36	β-glycosidic bond	GC, GPC, FT-IR, LC	Antioxidant, immunostimulatory		(14)

Gal, galactose; Rha, rhamnose; Ara, arabinose; Fuc, fucose; Glc, glucose; Man, mannose; Xyl, xylose; Man-N, epichitosamine; GlcA, glucuronic acid; Manp, mannopyranosyl; Xylp, xylose residue; GlcpA, glucuronic acid residue; GFC, gel filtration chromatography; TLC, thin-layer chromatography; IR, infrared spectra analysis; UV, UV-visible spectroscopy; FT-IR, Fourier Transform infrared spectroscopy; GPC, gel permeation chromatography; GC-MS, gas chromatography-mass spectrometry; HPLC, high performance liquid chromatography; PPC, paper chromatography; HPAEC-PAD, high-performance anion-exchange pulsed amperometric detection chromatography; COSY, correlation spectroscopy; TOCSY, total correlation spectroscopy; NOESY, nuclear overhauser enhancement spectroscopy; HMQC, heteronuclear multiple quantum correlation spectroscopy; HMBC, heteronuclear multiple bond correlation; HPGPC, high-performance gel permeation chromatography; SEM, scanning electron microscopy.

Diverse *T. aurantialba* polysaccharides were obtained because of different raw materials, extraction and purification methods. The molecular weight of *T. aurantialba* polysaccharides obtained shows diverse distribution, ranging from 14.0 kDa to 2924.6 kDa. Most of these polysaccharides are composed of more glucose (Glc), mannose (Man), xylose (Xyl), rhamnose (Rha) and galactose (Gal) with different molar fractions of the individual components. Remarkably, most fruit-body polysaccharides also contain uronic acid, which is considered to be highly correlated with polysaccharides activity. However, it is worth noting that most of the current studies use gas chromatography-mass spectrometry (GC-MS) to detect the uronic acid content in *T. aurantialba* (2, 4, 61, 73). To our best knowledge, this method is not suitable for the analysis of acidic sugars due to its low sensitivity to uronic acids. Moreover, the pre-column derivatization of PMP is unstable and prone to produce by-products. In fact, some new liquid chromatography-mass spectrometry techniques have been proposed to comprehensively characterize the composition of various monosaccharides such as uronic acid in polysaccharides, although most of these methods require derivatization, but with higher accuracy and sensitivity (75–78). In the future, these new technologies mentioned above can be applied to the study of *T. aurantialba* polysaccharides, so as to help us better analyze the structure-activity relationship of *T. aurantialba* polysaccharides and promote the application of *T. aurantialba* polysaccharides in medical and health care.

## 4 Biological activities

**Figure 2:**
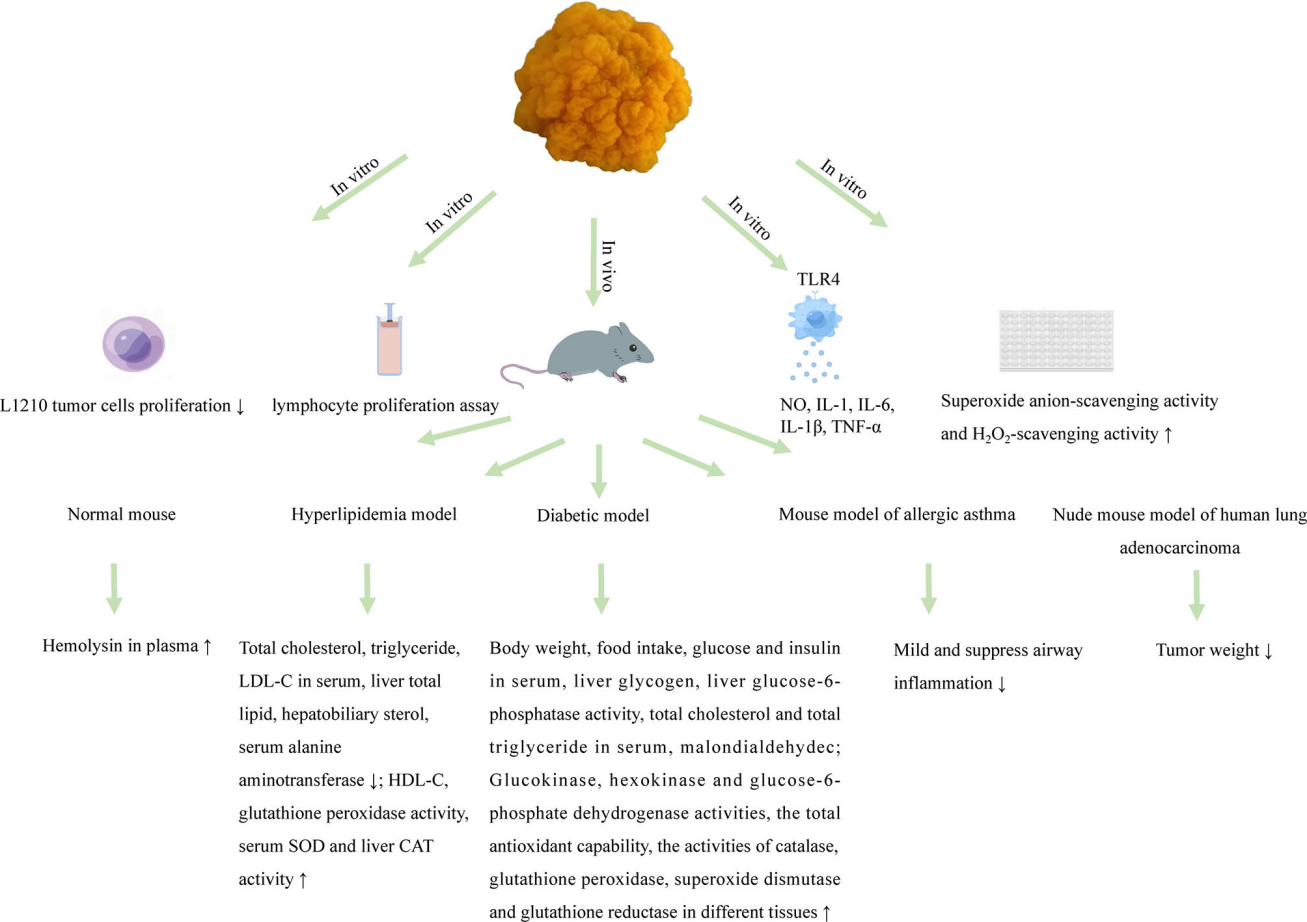
Biological activities of polysaccharides from *T. aurantialba*.

Polysaccharides obtained from *T. aurantialba* appear to be an all-round talent resisting a variety of chronic inflammatory diseases and protecting against different types of tumors, diabetes and cardiovascular diseases. Below is the summary of all the biological activities reported to date from the polysaccharides from this plant (**Figure 2**).

### 4.1 Immunostimulatory activity

The immunomodulatory activity of *T. aurantialba* polysaccharides is an exciting area that may yield new therapeutic avenues to maintain and improve human health. Macrophages are important members of the mononuclear macrophage system and exhibit various functions in immune response. A glucuronoxylomannan named TAP-3 extracted from the fruiting bodies of *T. aurantialba* could significantly stimulate the production of nitric oxide (NO), interleukin-1β (IL-1β) and tumor necrosis factor α (TNF-α) in RAW 264.7 macrophages, and anti-TLR4 (anti-toll-like receptor 4) treatment of TAP-3 stimulated macrophages could significantly reduce the levels of NO, IL-1β and TNF-α, suggesting that *T. aurantialba* polysaccharides might exert immunostimulatory activity through toll-like receptor 4 (TLR4) on macrophage surface (4). Du et al. used mouse splenic lymphocyte proliferation experiment to compare the immunostimulatory activity of crude polysaccharides, semi-purified polysaccharides and purified polysaccharides extracted from the fruiting bodies of *T. aurantialba*, and found that purified polysaccharide TAPA1 had the strongest immunostimulatory activity (64). Polysaccharide MCP extracted from *T. aurantialba* mycelium also had significant immunostimulatory activity and could promote the proliferation of macrophages, and stimulate macrophages to produce NO, interleukin-1 (IL-1), interleukin-6 (IL-6) and TNF-α (14).

### 4.2 Antitumor activity

So far, some studies have tried to use *T. aurantialba* polysaccharides in anti-tumor experiments. Yuan et al. found that oral or injection of *T. aurantialba* polysaccharides had inhibitory effect on transplanted human lung adenocarcinoma, and the antitumor activity of polysaccharides is stronger when injected (64). Choi confirmed that *T. aurantialba* polysaccharide, TAP2, had stronger anticancer activity by *in vitro* and *in vivo* experiments. Intraperitoneal injection of TAP could effectively inhibit the growth of malignant sarcoma cells (Sacroma 180) in BALB/C mice, with an inhibition rate of 73.3% (79). Lee et al. showed that intraperitoneal injection of crude polysaccharides from *T. aurantialba* could prolong the life span of mice with malignant sarcoma cell (Sacroma 180) cancer by 11.1% to 66.7%, which was consistent with Choi Pui-Yu’s research results (80). Zhang et al. reported that *T. aurantialba* polysaccharides could also inhibit the proliferation of L1210 tumor cells *in vitro* (17). These studies have only tentatively demonstrated the anti-tumor activity of *T. aurantialba* polysaccharides, and the specific molecular mechanism has not been reported. There is increasing evidence that complex signals from the tumor immune microenvironment have the potential to eliminate or promote malignancy. In addition, tumor immune microenvironment has become a new target for polysaccharides adjuvant therapy. However, whether *T. aurantialba* polysaccharides can reverse the tumor immune microenvironment by stimulating the immune system or enhancing the immune function of the body is worthy of further investigation.

### 4.3 Anti-diabetic activity

Long-term hyperglycemia will make the body in a long-term chronic inflammation state, directly leading to low immunity or immune dysregulation. Impaired immune function will in turn aggravate the occurrence and development of diabetes and its complications (81). Kiho et al. found that immune-stimulating agent *T. aurantialba* polysaccharide, TAP, had hypoglycemic effects on insulin-dependent diabetes mellitus (IDDM) model mice, non-insulin-dependent diabetes mellitus (NIDDM) model mice and glucose-loaded mice (73). In-depth study found that TAP could increase the activities of glucokinase and hexokinase involved in glucose uptake in the liver. At the same time, glucose-6-phosphatase activity, which was responsible for removing glucose from the liver, was decreased, suggesting that the hypoglycemic mechanism of TAP might be related to the accelerated glucose metabolism in the liver of diabetic mice (82). In addition, Kiho et al. also found that both TAP and acid hydrolysate TAP-H could reduce the plasma insulin level in diabetic mice, and the food intake and weight gain of mice fed TAP-H were less than those fed TAP, suggesting that TAP and TAP-H might have the effect of improving insulin resistance, and TAP-H was stronger than TAP. This might be related to the decreased viscosity and molecular weight of TAP-H or the enhanced activity after acid hydrolysis (83). Zhang et al. also reported that TMP, a polysaccharide from *T. aurantialba* mycelium, could significantly reduce blood glucose, serum triglyceride and total cholesterol levels and enhance insulin sensitivity index in rats with experimental type 、 diabetes mellitus, which might be related to the way that TMP increased insulin sensitivity and regulates lipid metabolism disorder (84). In addition, Deng et al. showed that exopolysaccharide of *T. aurantialba* also has hypoglycemic effects (15). However, it has not been reported whether *T. aurantialba* polysaccharides can affect the occurrence and progression of diabetes by intervening the immune-metabolic dialogue.

### 4.4 Hypolipidemic activity

The hypolipidemic effect of natural polysaccharides has been confirmed by many studies (85, 86), and a few studies have shown that *T. aurantialba* polysaccharides also have the potential to treat hyperlipidemia. TBE could increase the activities of superoxide dismutase, catalase, glutathione peroxidase and glutathione reductase, and reduced the content of malondialdehyde in the tissue of *T. aurantialba* fermentation broth extract, which indicated that TBE might play a role in correcting abnormal lipid metabolism, reducing oxidative stress and controlling diabetic complications (21). Wang et al. found that TMP, the mycelium polysaccharides of *T. aurantialba*, significantly increased HDL-C and HDL-C/TC ratio while reducing blood lipids, and effectively reduced LDL-C levels in the process of treatment, indicating that TMP had a certain role in the prevention and treatment of hyperlipidemia (18). Zhang et al. reported that mycelial polysaccharide TMP significantly reduced serum triglyceride and total cholesterol levels in type 、 diabetic rats, indicating that TMP effectively improved the lipid metabolism disorder in model animals and alleviated the toxic effects of hyperlipidemia (84). Kiho et al. showed that TAP and its acidolysis product TAP-H could significantly reduce plasma levels of total cholesterol and triglyceride. However, the potential lipid-lowering mechanism of *T. aurantialba* polysaccharides was still unclear (83).

### 4.5 Anti-asthmatic activity

Chinese medicine books mention that *T. aurantialba* can treat lung fever, phlegm, cold, cough, asthma, etc. (87). Jiang and Xiong showed that polysaccharides extracted from the fruiting bodies of *T. aurantialba*, TP, could reduce and inhibit airway inflammation in asthmatic rats, and had a significant protective effect on the delayed phase of allergic asthma model in rats (88). Besides, the significant protective effect of *T. aurantialba* polysaccharides on the rapid onset of allergic asthma was also found in the guinea pig allergic asthma model (89).

### 4.6 Hepatoprotective activity

After feeding polysaccharides extracted from the fruiting bodies or mycelium of *T. aurantialba* to rats with excess liver lipids for 8 weeks, total liver lipids and hepatobiliary sterols decreased by 55% and 50%, respectively. Besides, it can significantly reduce the serum alanine aminotransferase, protect and restore the acute and chronic liver poisoning caused by carbon tetrachloride, which indicated that *T. aurantialba* had a good effect on lowering lipids and protecting liver (90).

## 5 Structure-activity relationship analysis

**Figure 3:**
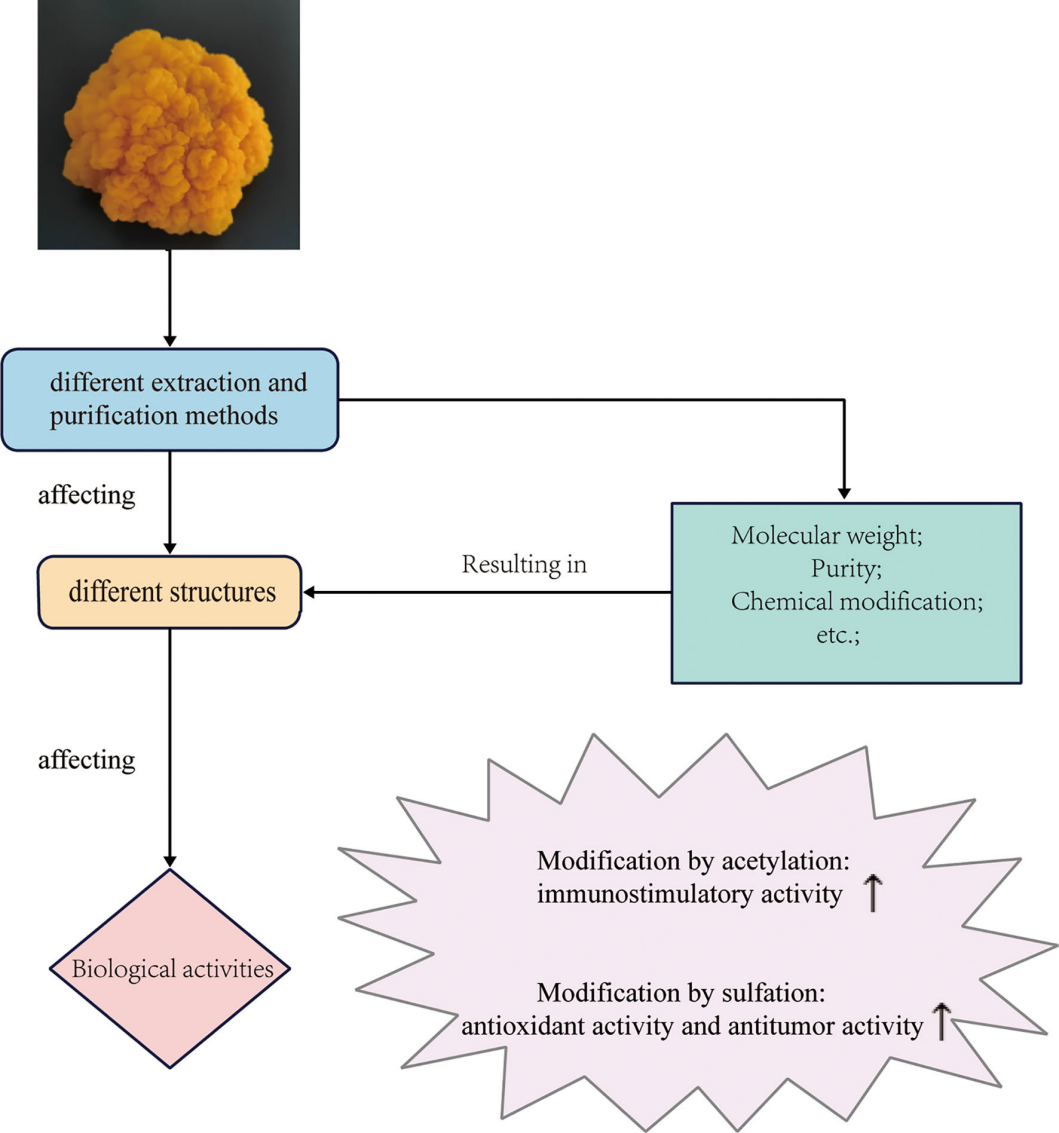
Structure-activity relationship of *T. aurantialba* polysaccharides.

It is generally believed that polysaccharides with different biological activities should have different structures (91). However, the studies on the structure-activity relationships of polysaccharides are limited due to their low purity and high heterogeneity. At present, only a few studies on the structure-activity relationship of *T. aurantialba* polysaccharides have been reported and some relationships can be inferred as follows (**Figure 3**). Yuan et al. used the purified polysaccharide TAP-3 from the fruiting bodies of *T. aurantialba* and its depolymerized small molecule fragments DTAP-3a and DTAP-3b to stimulate macrophages and found that the polysaccharide fragment DTAP-3b with the lowest molecular weight stimulated macrophages to produce the lowest levels of NO, IL-1β and TNF-α. This suggested that the immunostimulatory activity of polysaccharide TAP-3 was related to its molecular weight (4). Purified polysaccharide MCP from *T. aurantialba* mycelium had stronger immunostimulatory and antioxidant activities than crude polysaccharide CMCP before purification, which indicated that polysaccharide MCP activity might be related to purity (14). Zhang et al. reported that both TBE, the extract of *T. aurantialba* fermentation broth, and TMP, *T. aurantialba* mycelium extract, could regulate lipid metabolism and reduce oxidative stress, but the activity of TBE was significantly stronger than that of TMP, which might be related to the saponins in TBE (21). Zhao et al. demonstrated that the free radical scavenging activity of purified polysaccharide TAP was related to the pressure crushing time of *T. aurantialba* powder. The longer the crushing time, the more hydroxyl number of polysaccharides exposed, the stronger the DPPH and ·OH free radical scavenging activity (92). Chemical modification of polysaccharides such as acetylation and sulfation are an effective way to enhance the activity of natural polysaccharides (93–95). Du et al. compared *in vitro* immunostimulating activity of acid polysaccharide TAPA1 extracted from the fruiting bodies of *T. aurantialba* and its acetylated derivative TAPA1-ac and deacetylated derivative TAPA1-deac using the mouse spleen lymphocytes (MSLs) proliferation assay and nitric oxide (NO) production assay. The results showed that the stimulatory potency of MSLs of TAPA1-ac was higher than that of TAPA1, while the stimulatory potency of MSLs of TAPA1-deac was significantly lower than that of TAPA1 (96). Besides, Du et al. also found that TAPA1-s, a sulfated derivative of TAPA1, had more obvious immunostimulating activity than TAPA1 in the mouse spleen lymphocyte proliferation assay, indicating that sulfonation was an effective way to enhance the immunostimulating activity of polysaccharides (64). Zhang et al. compared the anti-tumor activity and antioxidant activity of *T. aurantialba* polysaccharide sulfated with different derivatization reagents, and the results showed that with the increase of the sulfate group substitution degree of *T. aurantialba*, its antioxidant activity and the activity of inhibiting tumor cell proliferation *in vitro* were enhanced (17). These studies indicated that the immunostimulatory activity and antitumor activity of *T. aurantialba* polysaccharides were affected by the types of functional groups and the degree of side chain branching. Further chemical modifications should be investigated to explore the effects of chemical modifications on other biological activities of *T. aurantialba* polysaccharides. Understanding the clear structure-activity relationship is the premise for the industrial production of active polysaccharides from *T. aurantialba* and the development of safe and effective new drugs. In the future, different extraction, separation and purification methods should be used to obtain more polysaccharides with different structures, and the application of various derivatization methods should be strengthened to carry out more studies on the relationship between structure and activity.

## 6 Application

**Figure 4:**
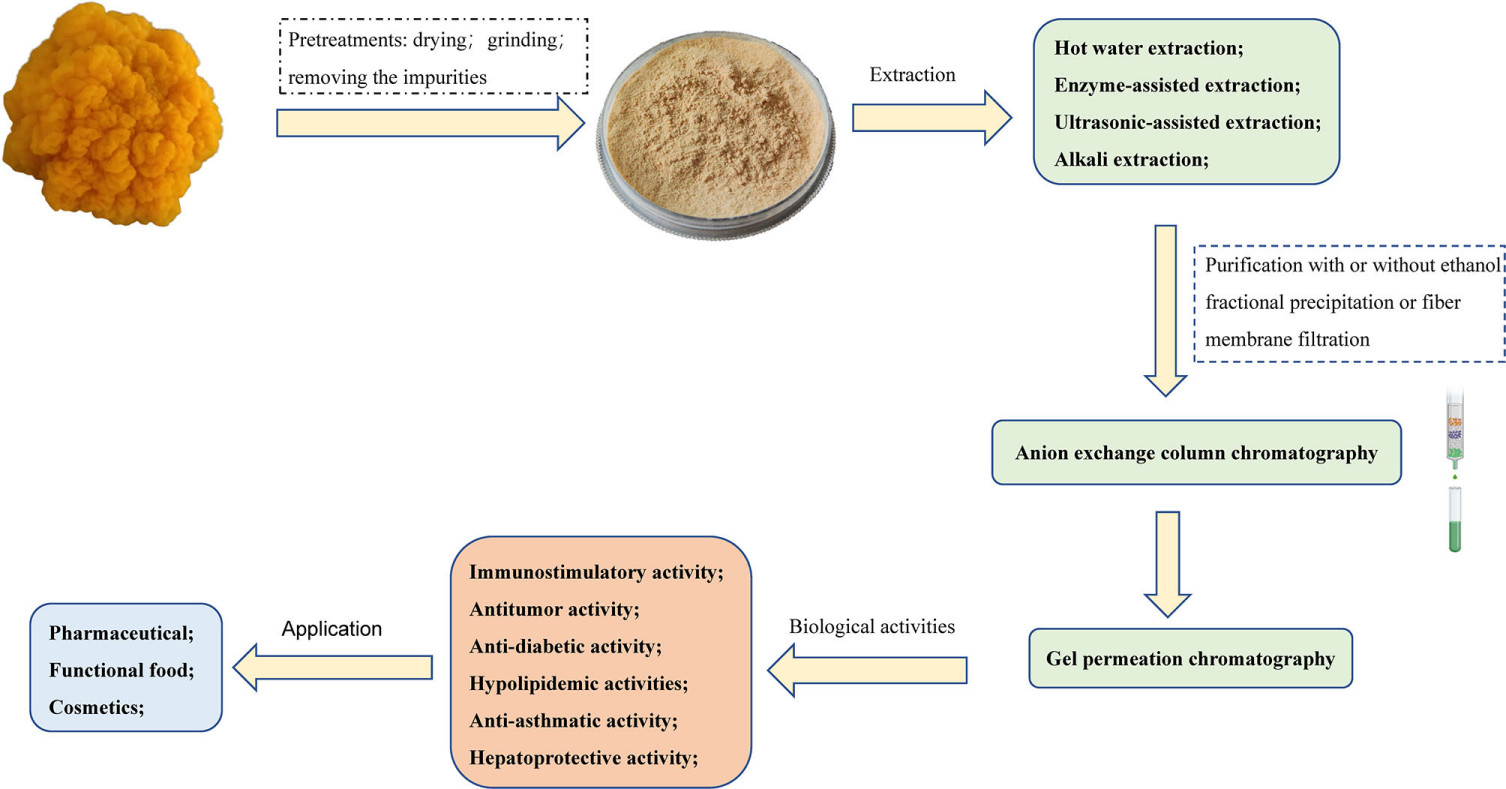
Schematic representation of the extraction, purification, biological activities and applications of *T. aurantialba* polysaccharides.

Due to its diverse biological activity and special taste, *T. aurantialba* polysaccharides has been widely used in medicine, food, skin care and other industries. *T. aurantialba* glycopeptide capsules made from *T. aurantialba* polysaccharides have shown clear hepatoprotective effects in preliminary clinical trials and phase II clinical trials, without side effects, and can assist the treatment of liver diseases (97, 98). Pharmacological studies have showed that *T. aurantialba* glycopeptide capsules may mainly prevent hepatitis and other diseases by protecting hepatocytes, regulating or stabilizing cell functional activities, and improving immunity (99). Polysaccharides from *T. aurantialba* also can be used as food additives in bread production. For example, when 1% to 3% *T. aurantialba* crude polysaccharides is added to the flour, the colloid property of the dough can be greatly improved, and the bread taste is soft and delicate, with regular large holes and uniform organization (100). The nutritional liquid made from *T. aurantialba* polysaccharides is a kind of health drink with novel taste and bright color, which not only has good immune-promoting effect, but also has no toxic and side effects (101). The drink made from the fermentation broth of *Tremella aurantialba* is also a health drink with unique taste and immune and anti-inflammatory effects (102). In addition, *T. aurantialba* polysaccharides have lubricity, film-forming property, moisturizing property and anti-radiation activity (103, 104), thus it has been used to make facial masks, facial masks, moisturizers and other high-end skin care and beauty cosmetics (104–106). Patents list of products containing *T. aurantialba* polysaccharides is shown in **Table 3**. **Figure 4** shows the methods of extraction, purification, biological activities and applications of the polysaccharides obtained from *T. aurantialba*.

**Table 3:** Application of *T. aurantialba* polysaccharides.

Application	Main composition	Pharmacological properties	Publish number
Toast bread	Mycelia polysaccharides of *T. aurantialba*, high-gluten flour, *T. aurantialba* Fungus Paste, diced *T. aurantialba*, sugar, butter, eggs, active dry yeast, salt, milk powder	Enhancing taste and reducing the loss of heat-sensitive nutrients	CN202111282650.6
Compounded combinations	Degradation products of *T. aurantialba* polysaccharide, glycyrrhizic acid monoammonium salt n-hydrate	Moisturizing	CN202111180573.3
Compounded combinations	Crude Polysaccharide of *T. aurantialba*, glucosyl hesperidin	Protecting, soothing and repairing blue light-induced skin cell damage	CN202111271004.X
Chewable tablets	*T. aurantialba* polysaccharides, edible starch, lactose, sucrose, mannitol, magnesium stearate, edible silica gel	Enhancing immunity function	CN201910973358.5
Face Mask	*T. aurantialba* polysaccharides, rose puree, extract of grape seed, sodium hyaluronate, deionised water	Skin whitening and moisturizing	CN201811252114.X
Humectants	*T. aurantialba* polysaccharides, wheat protein hydrolysate, vitamin E acetate, rose essence, methylisothiazoline, glycerin	Hydrating skin and anti-aging	CN201210554360.7
Jelly	*T. aurantialba* concentrate and *Tremella fuciformis Berk.* fruits, kiwi fruit juice, honey, kodran polysaccharide	–	CN200910155166.X
Yogurt	*T. aurantialba* fruiting bodies extract or *T. aurantialba* mycelia concentrate, fresh milk, milk powder, fruit juice, water, lactic acid bacteria	Anti-aging, anti-inflammation and anti-radiation	CN200710053545.9
Functional beverage	*T. aurantialba* concentrate, flower juice, seasoning, xanthan gum	–	CN201410408378.5

## 7 Conclusion and perspectives

According to the traditional Chinese medicine and modern pharmacological research, *T. aurantialba* has high medicinal value and nutritional value. Polysaccharide is one of the major active components of *T. aurantialba*, which is abundant in *T. aurantialba*, and has significant immune-stimulating activity and the ability to resist various inflammatory diseases. In recent years, the isolation methods, structural properties, biological activities and applications of *T. aurantialba* polysaccharides have been widely reported. However, much remains to be explored in the field of *T. aurantialba* polysaccharides. Firstly, efficient enzyme extraction and ultrasound-assisted extraction methods have been proposed by a few studies for the extraction of *T. aurantialba* polysaccharides, but there is a lack of comprehensive and detailed comparison with hot water extraction. New separation techniques such as microwave-assisted extraction, high pressure pulse extraction, supercritical fluid extraction can be further used in the extraction process of *T. aurantialba* polysaccharides, thereby improving the yield and activity of polysaccharides. Second, the structure of polysaccharides is affected by the conditions of separation and purification, the source of samples and other factors. At present, there are few studies on the structure of *T. aurantialba* polysaccharides, especially the advanced structure. Future research should be combined with advanced and convenient structure characterization technology to deeply explore the structural information of *T. aurantialba* polysaccharides. Third, as the main active component of *T. aurantialba*, the bioactivities of *T. aurantialba* polysaccharides is not fully explore, especially the specific molecular mechanism such as immunostimulating activity is rarely reported. Various *in vitro* and *in vivo* experiments and clinical trials should be supplemented to improve the research results. Fourth, there are few studies on structure-activity relationship of *T. aurantialba* polysaccharides and structural modification techniques such as hydroxymethylation, selenylation and phosphorylation can be further applied to the activity study of *T. aurantialba* polysaccharides. In the future, it is hoped that the research on *T. aurantialba* polysaccharides can be more comprehensive and thorough, and the standardized preparation technology of *T. aurantialba* polysaccharides will be established as soon as possible, so as to realize the industrial production and application of *T. aurantialba* polysaccharides.

## Glossary

**Table:**

IR	infrared spectroscopy
HPAEC-PAD	high-performance anion-exchange pulsed amperometric detection chromatography
GC-MS	gas chromatography-mass spectrometry
NMR	nuclear magnetic resonance spectroscopy
HPLC	high-performance liquid chromatography
GC	gas chromatography
GPC	gel permeation chromatography
UV	ultraviolet
Glc	glucose
Man	mannose
Xyl	xylose
Rha	rhamnose
Gal	galactose
NO	nitric oxide
IL-1b	interleukin-1b
TNF-a	tumor necrosis factor a
anti-TLR4	anti-toll-like receptor 4
TLR4	toll-like receptor 4
IL-1	interleukin-1
IL-6	interleukin-6
IDDM	insulin-dependent diabetes mellitus
NIDDM	non-insulin-dependent diabetes mellitus
HDL-C	high density liptein cholesterol
TC	serum total cholesterol
LDL-C	low-density lipoprotein cholesterol
MSLs	mouse spleen lymphocytes
HWE	hot-water extraction
UAE	ultrasoundassisted extraction
EAE	enzyme-assisted extraction
ABE	alkali-base extraction
Ara	arabinose
Fuc	fucose
Man-N	epichitosamine
GlcA	glucuronic acid
Manp	mannopyranosyl
Xylp	xylose residue
GlcpA	glucuronic acid residue
GFC	gel filtration chromatography
TLC	thin-layer chromatography
FT-IR	Fourier Transform infrared spectroscopy
PPC	paper chromatography
COSY	correlation spectroscopy
TOCSY	total correlation spectroscopy
NOESY	nuclear overhauser enhancement spectroscopy
HMQC	heteronuclear multiple quantum correlation spectroscopy
HMBC	heteronuclear multiple bond correlation
HPGPC	highperformance gel permeation chromatography
SEM	scanning electron microscopy
